# The host immune response to *Mycobacterium tuberculosis* determining protection or disease progression

**DOI:** 10.1038/s41590-026-02529-z

**Published:** 2026-05-18

**Authors:** Margarida Saraiva, William J. Branchett, Jyothi Rengarajan, Anne O’Garra

**Affiliations:** 1 i3s, Instituto de Investigação e Inovação em Saúde, Porto, Portugal; 2 Laboratory of Immunoregulation and Infection, The Francis Crick Institute, London, UK; 3 Emory Vaccine Center, Emory National Primate Center, Emory University, Atlanta, GA, USA; 4 Division of Infectious Diseases, Department of Medicine, Emory University School of Medicine, Atlanta, GA, USA

## Abstract

Tuberculosis (TB) remains to this day a global health issue and leading cause of death. A deeper understanding of the immune response to *Mycobacterium tuberculosis* infection is critical for the design of effective vaccines, better therapies and faster diagnosis. Although immune factors involved in protection against TB have long been identified, these cannot predict the efficacy of TB vaccines, nor which individuals will control the infection or progress to active TB disease. This is especially challenging given the complexity of *M. tuberculosis* infection states and the breadth of TB disease severities, which is increasingly apparent from basic, clinical and translational research. Here, we discuss the evolving spectrum of *M. tuberculosis* infection outcomes and TB disease and how the host immune response determines and unfolds across this spectrum, from susceptibility to resistance to disease. We also discuss how the immune response and TB outcomes are modulated by the natural diversity of *M. tuberculosis*. Integration of these novel concepts with fast-evolving systems immunology approaches holds great potential to advance our understanding of the immune responses to *M. tuberculosis* to improve TB control.

## Introduction

Tuberculosis (TB), mostly caused by *Mycobacterium tuberculosis* has claimed more lives than any other infectious disease in history and, despite over a century of vaccination and decades of antibiotic therapy, it remains a leading cause of death ^1^. Although the majority of individuals infected with *M. tuberculosis* remain healthy, there are annually 10.7 million new cases of active TB disease and 1.2 million deaths ^1^. Several key problems hamper TB control. The only currently licensed vaccine, Bacille Calmette-Guerin (BCG), offers poor protection against pulmonary TB in children and adults ^2^. For a breakthrough in TB vaccine development, improved knowledge on how, when and where to trigger a protective immune response against TB is required. TB diagnosis relies mainly on detection of *M. tuberculosis* in sputum of suspected patients which is often difficult to achieve ^1^. As a result, the time between disease onset and diagnosis is typically long and a large number of people with TB remain undiagnosed, both issues increasing the likelihood of transmission. Current antibiotic treatment of TB relies on administration of several antibiotics for at least 6 months ^1, 3^, with undesirable side-effects, and the existence of resistant or multidrug-resistant *M. tuberculosis* strains can delay cure ^1^. The development of host-directed therapies holds the potential to shorten and improve treatment regimens. This has proven a difficult task, given the complexity and our incomplete understanding of the immune responses associated with either protection or disease progression in TB. Additionally, recent epidemiologic, clinical and experimental data on TB and *M. tuberculosis* infection outcomes clearly illustrate that TB is not a single disease, but instead a spectrum of infection and disease states ^4^. Finally, the genetic diversity of circulating *M. tuberculosis* bacteria and their high adaptation to humans ^5^ contribute to the success of *M. tuberculosis* as a pathogen and make it difficult to fully understand the breadth of host interactions and responses during infection. The genetic diversity of *M. tuberculosis* has been classically neglected in TB research, because it is overall very low ^6^. This is due to a low mutation rate, slow replication time and robust, high-fidelity DNA repair mechanisms that together preserve genomic stability. However, it is now well-established that small genetic differences in *M. tuberculosis* impact immunity, transmission and disease severity. A greater understanding of the host immune response to diverse *M. tuberculosis* strains and across the TB spectrum may provide strategies to improve TB control through effective vaccines and host-directed therapies. In this review, we discuss the most recent advances in our knowledge of the immune response shaping TB outcomes, focusing on critical host and pathogen traits, and their interactions, including reports from human studies.

### States of *M. tuberculosis* infected individuals from asymptomatic to progression to active TB

Past or current infection with *M. tuberculosis* is routinely tested by measuring T cell responses to mycobacterial antigens either in the tuberculin skin test (TST) which also detects prior BCG vaccination, or by the *M. tuberculosis* antigen-specific interferon (IFN)-γ release assays (IGRA). However, neither test can distinguish current from previously cleared *M. tuberculosis* infection, nor can they predict who will remain healthy or those who will progress to active TB disease. Individuals with TST or IGRA positive tests in the absence of clinical symptoms or signs of TB disease are classically categorized as having latent TB infection (LTBI) ^1, 3, 4^. Individuals who have been exposed to *M. tuberculosis* and do not go on to develop TB disease, may remain IGRA negative or may become IGRA positive, in some cases with small lung inflammatory changes or immune responses in lymph nodes, likely requiring a highly sensitive positron emission tomography–computed tomography (PET-CT) scan for detection based on metabolic activity ^7^. On the other hand, *M. tuberculosis* infected individuals who go on to develop TB disease, may at baseline be IGRA negative or positive, and will become IGRA positive and develop radiological changes in their lungs as they progress to symptomatic TB ^8, 9, 10^. Infected individuals may also present as asymptomatic TB, previously referred to as subclinical TB ^1^, characterized routinely in the clinic as being either microbiologically positive, with chest X-ray changes, or both ^4^. Whether all individuals with asymptomatic TB progress to symptomatic clinical TB is not known.

Although it had been thought that 5-10% of *M. tuberculosis* infected asymptomatic individuals could develop TB by reactivation at any stage in their lifetime ^11^, more recent studies show that the majority of infected individuals who will progress to active TB will do so within 1 – 3 years post-infection ^11, 12, 13^, although reactivation at later stages may occur, albeit less frequently ^11, 12^. It should be noted that apparent TB reactivation could reflect a new infection, confounding tracking of disease progression and studies of the immune response in humans, particularly in high burden TB settings ^14, 15^.

**Figure 1:**
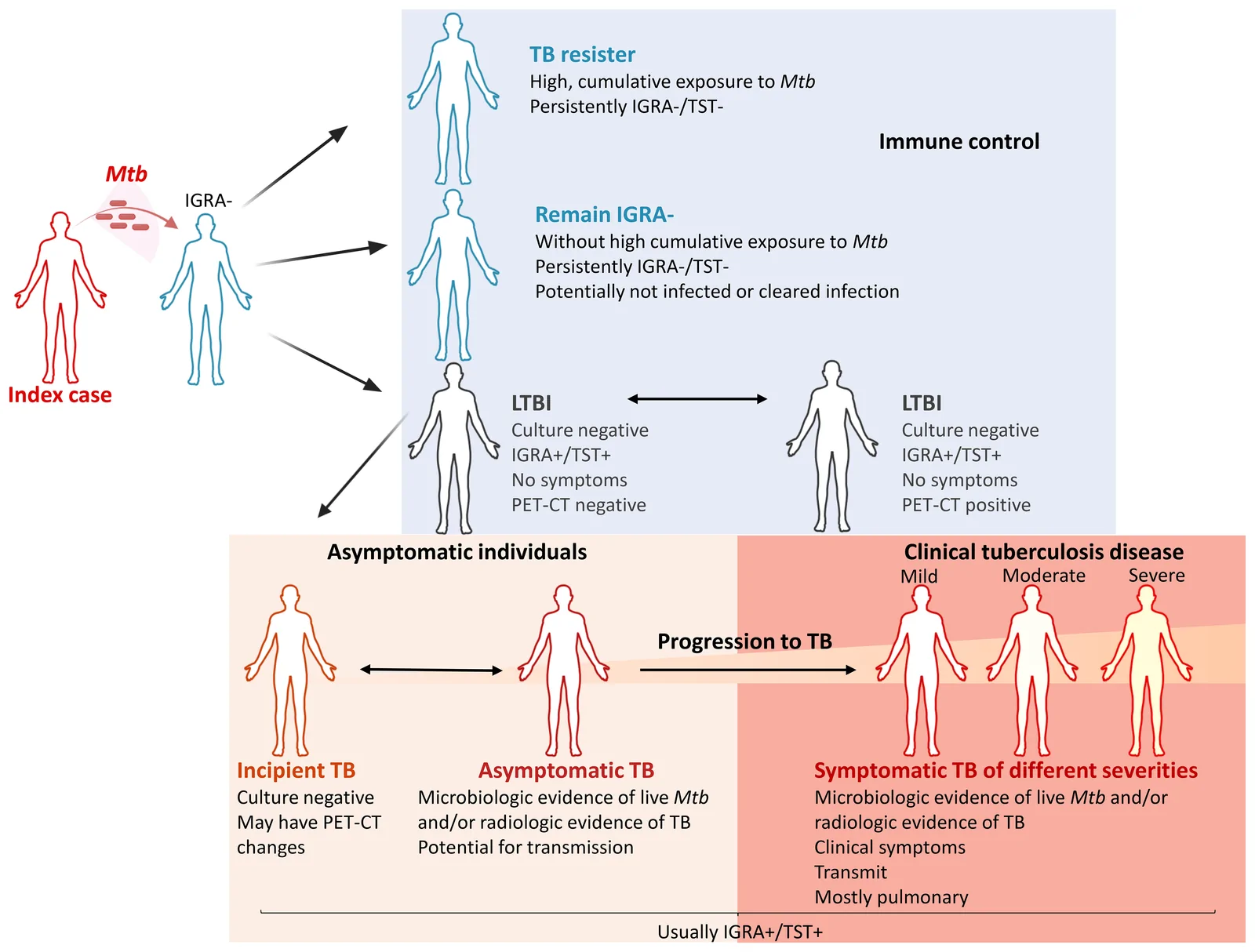
The TB spectrum. Transmission of *M. tuberculosis* occurs via aerosol, from index cases to their contacts. Different outcomes of infection are possible, being defined by the detection of bacteria in culture, PET-CT or radiologic changes, adaptive memory measured by TST or IGRA tests and presence of symptoms. The vast majority of individuals achieves immune control of the infection, with three main described groups: TB resisters, who remain IGRA/TST negative despite persistent exposure to *M. tuberculosis* ^18^; those who also remain IGRA/TST negative without cumulative exposure to *M. tuberculosis*; and those with latent TB infection (LTBI), who become IGRA/TST positive but do not develop TB disease ^16, 17^. A minority of individuals (estimated to be 5-10%) however do progress to TB disease, usually displaying IGRA/TST positive tests. Incipient and asymptomatic TB groups are composed by individuals who remain asymptomatic but present PET-CT/radiologic changes ^4, 9^. Eventually, symptomatic TB of different severities may develop, with microbiologic, radiologic and clinical evidence of TB disease ^1, 3^. It is now considered that transmission may occur from both asymptomatic and symptomatic TB patients ^20^. *IGRA, interferon gamma release assay; LTBI, latent tuberculosis infection; Mtb, Mycobacterium tuberculosis; PET-CT, positron emission tomography–computed tomography; TB, tuberculosis; TST, tuberculin skin test*.

Binary classification of TB into active and latent disease no longer reflects our understanding of the disease ^16, 17^, with a much more complex picture emerging (Figure 1). The spectrum of TB infection and disease also encompasses individuals who, despite heavy and repeated exposure to TB-causing bacteria, resist infection and remain IGRA negative, termed resisters ^18^, and those who go on to become IGRA positive and either remain healthy or progress to TB disease. Asymptomatic TB cases may progress to TB disease and may contribute to transmission from a very early stage ^4, 19, 20^. Indeed, advanced studies on the aerobiology of TB are showing that release of *M. tuberculosis* aerosols does not necessarily require cough, with tidal breathing being sufficient ^21^, irrespective of TB diagnosis ^22^. This was supported by studies using face-mask sampling to detect exhaled *M. tuberculosis* by asymptomatic individuals ^23, 24^. Active TB most frequently presents as pulmonary TB with varying disease severity, with the most severe cases presenting with extensive necrosis and lung cavitation ^25, 26, 27^. *M. tuberculosis* can disseminate from the lung to lymph nodes and other organs, however the impact of dissemination on disease progression and immunity remains incompletely understood ^28^. *M. tuberculosis* dissemination may result in extra-pulmonary TB which accounts for up to 15% of TB cases world-wide ^26, 29, 30^, with the most lethal and disabling form being tuberculous meningitis ^31^. This review will primarily focus on the immune response in pulmonary tuberculosis.

### An overview of the immune response and pathology in TB

The current view of events leading to the development of the immune response during *M. tuberculosis* infection has been mainly drawn from studies using animal models and is extensively reviewed elsewhere ^26, 32, 33, 34^. In brief, once inhaled, *M. tuberculosis* travels through the airway and in the alveoli initially infects alveolar macrophages. Alveolar macrophages not only support *M. tuberculosis* growth, but also transit to the lung parenchyma, where the bacilli disseminate to other phagocytes, including interstitial macrophages, incoming monocyte-derived macrophages, monocytes, neutrophils and dendritic cells (DCs). Infected DCs eventually migrate to the lymph nodes, where they prime antigen-specific CD4 T cells to proliferate and differentiate, which are then recruited back to the lung. IFN-γ-producing CD4 T_H_1 cells are considered key for controlling *M. tuberculosis* with IL-17-producing T_H_17 cells potentially also playing a role. Central immune mediators protective against *M. tuberculosis* infection are IL-12/IFN-γ axis and TNF which are common to humans and animal models ^32, 33, 34, 35, 36^, with IL-1 shown to be protective in mouse models ^37, 38^. In humans who develop TB disease, the immune response to *M. tuberculosis* culminates in the formation of TB granulomas in the lung. Granulomas are hallmark structures that are highly heterogeneous even in the same individual, with some granulomas restricting *M. tuberculosis*, and others necrotizing and leading to bacterial proliferation, tissue damage and disease, thus promoting transmission to other hosts ^25, 39^. However, pharmacological disruption of the granuloma structure may be a strategy improve the access of TB drugs to the areas with higher bacterial burden and perturb the physiological adaptations of *M. tuberculosis* to the different niches (reviewed in ^40^).

Our knowledge of the immune response and pathogenesis of pulmonary TB relates mainly to primary TB, i.e., to what happens when an individual is infected with *M. tuberculosis* for the first time without prior immunity to infection. Although less studied, most cases of TB in humans are post-primary TB, which occurs in the context of pre-existing immunity. This results in lesions that are distinct from those seen in primary TB, as shown in a recent study of the pathology of lung TB lesions from the pre-antibiotic era ^41^. A major reported difference is that in primary TB granuloma formation starts in the lung interstitium, whereas in post-primary TB granuloma formation starts as pneumonia in the alveoli ^41^.

### Genetic susceptibility to TB disease

**Figure 2:**
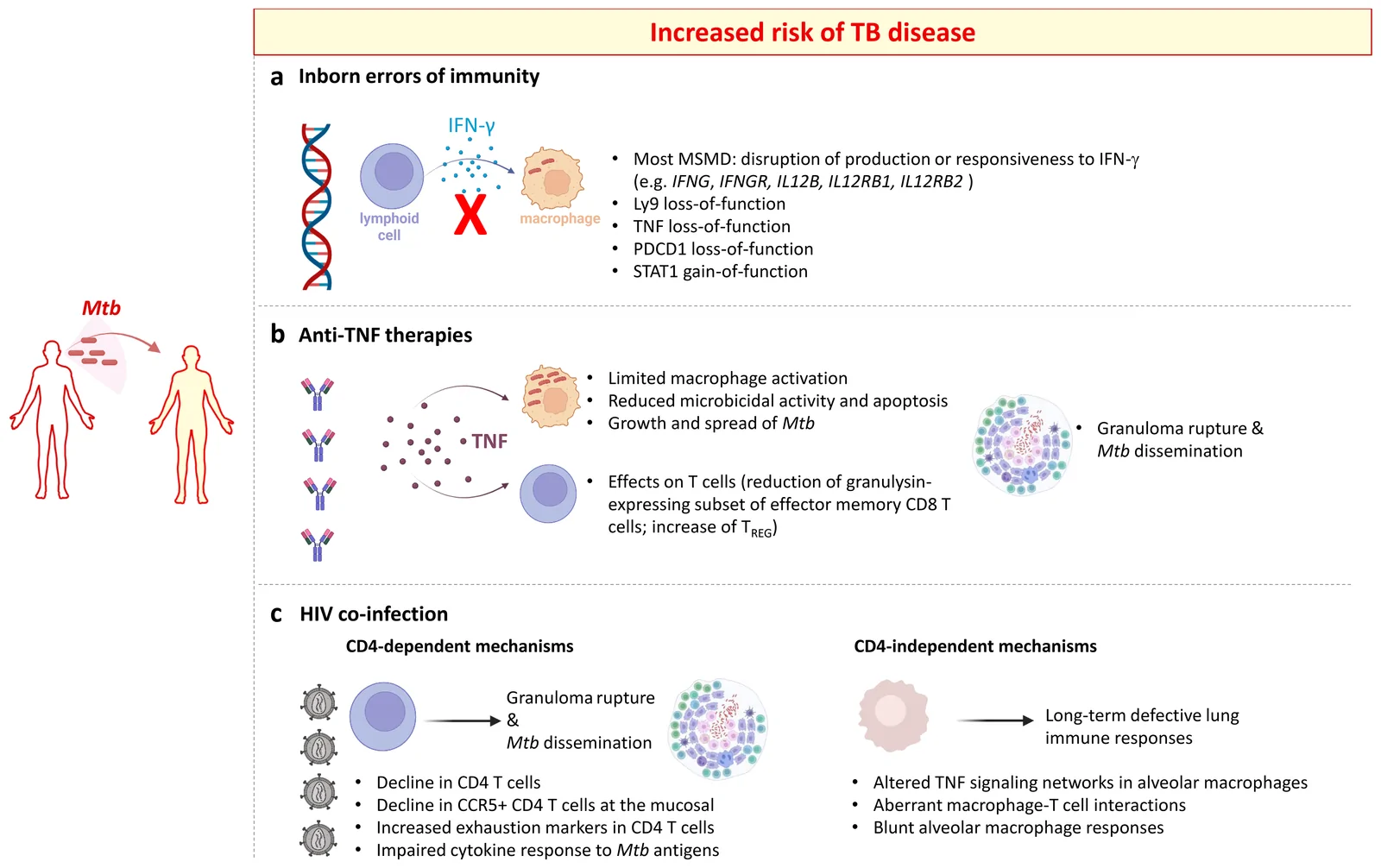
Mechanisms of TB susceptibility. Several conditions increase the individual risk of developing TB disease upon infection with *M. tuberculosis*. **a**. Inborn errors of immunity, in particular Mendelian susceptibility to Mycobacteria disease (MSMD), have been highly studied over the years. Most MSMD result from inborn errors of IFN-γ immunity, affecting the production or the response to this cytokine and impairing mycobacteria control by phagocytes ^35^. The study of MSMD has greatly contributed to the understanding of the immune mechanisms required for protection during *M. tuberculosis* infections. **b**. Deregulation of TNF responses through anti-TNF therapies impacts optimal macrophage and T cell responses to *M. tuberculosis* and promotes granuloma disorganization with subsequent *M. tuberculosis* dissemination, thus compromising the pathogen’s control ^36, 50^. **c**. Co-infection with HIV impairs CD4 T cell dependent and independent protective responses leading to increased susceptibility to TB and representing the highest individual and populational risk for TB disease ^10, 51^. *IFN, interferon; MSMD, Mendelian susceptibility to Mycobacteria disease; Mtb, Mycobacterium tuberculosis; TB, tuberculosis; TNF, tumour necrosis factor; TREG, regulatory T cell*.

Direct evidence of genetic susceptibility to TB (Figure 2a) is provided by studies on Mendelian susceptibility to Mycobacterial disease (MSMD) ^35^. This is a group of rare, low penetrance loss-of-function genetic mutations affecting transcription factors, intracellular signaling mediators or cell surface receptors that broadly compromise IFN-γ responses ^35^. The etiology of most MSMD relates to impairment of the IL-12/IFN-γ axis, which is also fully supported by studies in animal models of *M. tuberculosis* infection ^32, 33, 34^. The continuous study of MSMD, combined in some cases with mouse models of *M. tuberculosis* infection, has revealed novel mechanisms underlying the regulation of optimal IFN-γ production by immune cells, including a role for TYK2 and IL-23, the transcription factor RORγt and also ISG15 ^42, 43, 44, 45, 46^. Other inborn errors of immunity associated with susceptibility to TB have been described, including gain-of-function in STAT1 ^47^ and loss-of-function in Ly9 ^48^ or TNF ^49^. A genetic basis for TB susceptibility grounded on TNF further supports the protective role of this molecule as previously described in patients receiving anti-TNF therapies ^36^ (Figure 2b) and in TNF deficient mice ^50^.

### Susceptibility to TB caused by immunosuppression

The condition that most markedly increases the risk of TB at the individual and population levels is co-infection with human immunodeficiency virus (HIV) ^10, 51^ (Figure 2c). People with HIV present a general immunosuppression, with the characteristic decline of CD4 T cells, which significantly increases their risk of progressing to TB disease ^52^. Particularly important, is the early depletion of effector memory CCR5+ CD4 T cells at the mucosal sites, as informed by experiments performed in *M. tuberculosis*/simian immunodeficiency virus (SIV)-coinfected non-human primates (NHP) ^53^. Additionally, CD4 T cells from people living with HIV (PLWH) exhibit increased exhaustion markers, and those from people with HIV-TB co-infection display impaired cytokine responses to *M. tuberculosis* antigens (reviewed in ^51^). In individuals with advanced HIV infection, TB granulomas lose their typical cellular organization, which reflects the characteristic clinical manifestation of extrapulmonary, disseminated TB ^51^. Ultimately, as a result of multiple immune alterations, people with HIV-TB coinfection present granulomas with lower numbers of CD4 T cells, alterations in macrophage activation and maturation and enhanced neutrophil infiltration (reviewed in ^10^).

Notably, even after successful antiretroviral therapy (ART) that suppresses viral load and restores CD4 T cell counts, PLWH still present a 4-fold higher risk of developing TB as compared to people without ^54^. This suggests that treated HIV infection imposes long-term defective lung immune responses to *M. tuberculosis*, irrespective of CD4 T cell restoration. This is also supported by studies in *M. tuberculosis*/SIV coinfection, where SIV was shown to disrupt protective immune responses against latent *M. tuberculosis* infection beyond the loss of CD4 T cells ^55^. A recent study analysed the single cell transcriptome of bronchoalveolar lavage cells from PLWH on ART upon *in vitro* infection with *M. tuberculosis* and revealed impaired TNF signaling networks in alveolar macrophages from PLWH compared to healthy HIV uninfected controls, along with aberrant macrophage-T cell interactions ^56^. In another study, alveolar macrophages from PLWH showed blunted transcriptional, epigenetic and cytokine responses to *M. tuberculosis* infection as compared to controls ^57^. In the NHP model of *M. tuberculosis*/SIV coinfection the timing of ART administration impacted LTBI reactivation, with earlier ART leading to reduced granuloma formation and improved tissue pathology. This impact of early ART therapy corelated with robust CD8 T effector memory responses, although only partially restoring CD4 T cells in the lungs ^58^, therefore suggesting a key role for early events of SIV coinfection in shaping LTBI reactivation.

Beyond HIV, several other TB risk factors have been described that negatively impact host immune responses and present as TB co-morbidities (Table 1).

### Determinants of host resistance to TB disease

**Figure 3:**
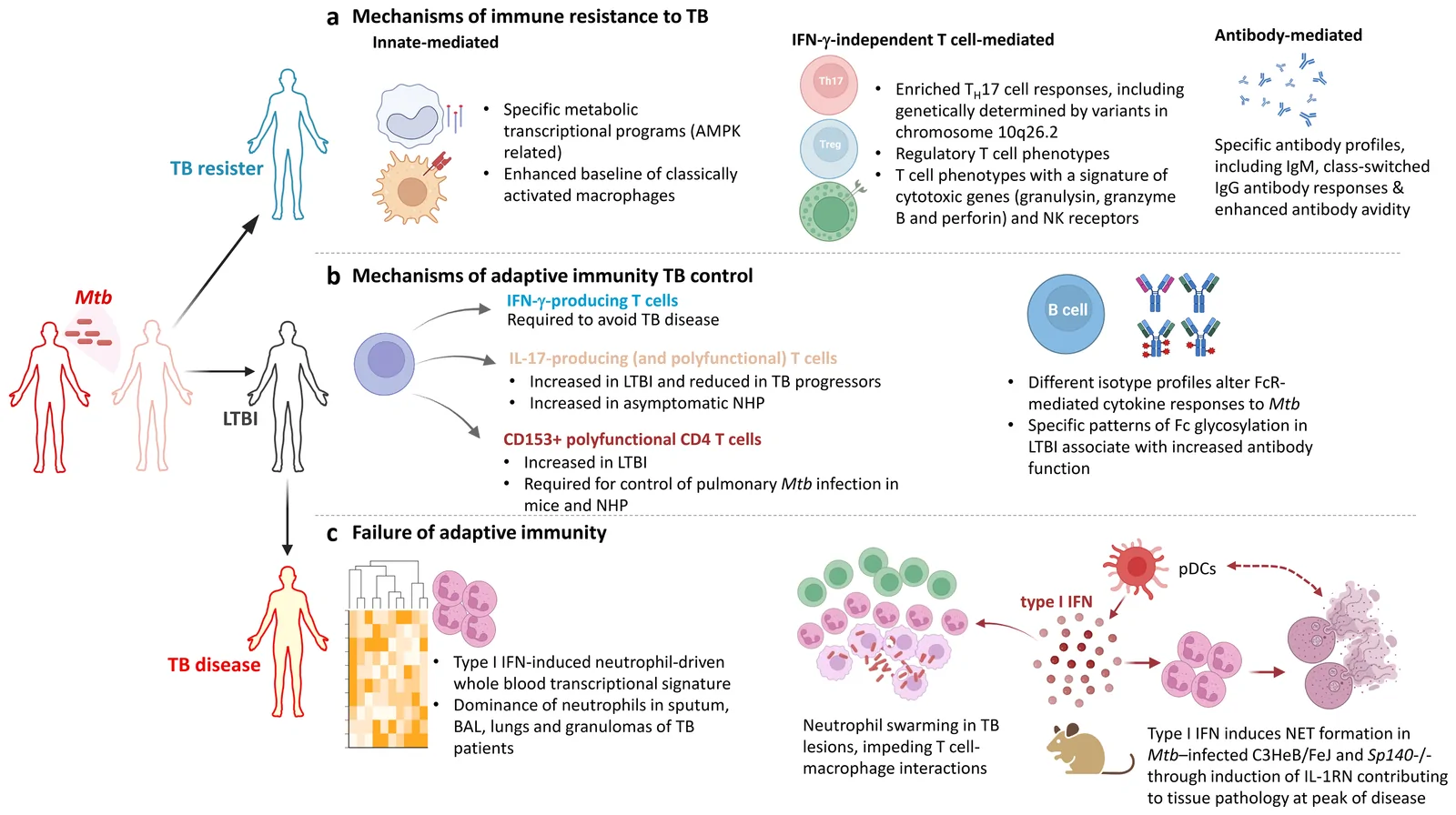
Immune mechanisms dictating immune control or TB disease. **a**. TB resisters do not convert to IGRA/TST positive tests nor develop TB disease, despite being persistently exposed to *M. tuberculosis* ^18^. Different immune mechanisms have been described in TB resisters, including potent innate responses ^76^, effective T cell responses that are IFN-γ-independent ^77, 81^, and specific protective antibody profiles ^82^. **b**. Immune control of *M. tuberculosis* is also achieved in the LTBI group, albeit through different mechanisms. In this case, T cell protective responses are mediated not only, but also by IFN-γ ^93, 94, 97, 100, 101^. Specific antibody profiles, including specific patterns of Fc glycosylation, have also been associated with LTBI ^109, 110^. **c**. Failure of adaptive immunity leads to TB disease. A type I IFN-induced neutrophil-driven transcriptional signature has been identified in TB patients ^114^ and a pronounced neutrophilia is characteristic of TB disease ^121^. Recent studies in mouse models of *M. tuberculosis* are shedding light into the mechanisms linking type I IFN, neutrophils and failure of adaptive immunity and tissue pathology ^128, 132, 133^. Importantly, the spatial positioning of neutrophils within TB lesions impairs T cell-macrophage interactions needed for *M. tuberculosis* control ^124, 125^. *BAL, bronchoalveolar lavage; IFN, interferon; IL-1RN, interleukin 1 receptor antagonist; LTBI, latent tuberculosis infection; Mtb, Mycobacterium tuberculosis; NET, neutrophil extracellular trap; NHP, non-human primate; NK, natural killer cell; pDC, plasmacytoid dendritic cell; TB, tuberculosis; TH, T helper cell; -/-, deficient*.

Initial studies performed in household contacts of people with TB disease showed that a proportion of individuals repeatedly exposed to TB do not become TST positive, suggesting that natural resistance to *M. tuberculosis* infection may exist ^69^. Resistance to TB disease is now acknowledged as an important outcome of *M. tuberculosis* infections, supported by several epidemiologic studies in different settings ^18, 70^. The current definition of resisters comprises individuals persistently exposed and do not convert to PPD/IGRA positivity. Presumably, these individuals are exposed to cumulative doses of *M. tuberculosis*. However, there may be a threshold exposure beyond which “resistance” becomes unlikely/impossible since there is evidence that high and repeated exposure of prisoners with *M. tuberculosis* results in a greater probability of IGRA conversion and TB disease ^71^. Understanding the immune events underlying individuals classified as resisters (Figure 3a) is fundamental to uncover the protective correlates of *M. tuberculosis* infection and is of great importance for the development of vaccines and host-directed therapies.

Family studies have provided strong evidence for the central role of genetics in resistance to *M. tuberculosis* infection and TST reactivity ^72, 73, 74, 75^. More recently, several functional mechanisms have been proposed to explain TB resistance. An obvious explanation would be the clearance of *M. tuberculosis* by innate immune mechanisms, which may operate in TB resisters. This “innate resistance” is supported by the description of a specific monocyte metabolic transcriptional program in TB resisters, which was distinguished from LTBI by free-fatty acid transcriptional programs and by genetic variation in AMPK, a central metabolic regulator ^76^. T cell-dependent resistance mechanisms have also been proposed, although mostly independent of IFN-γ ^77^. Instead, T_H_17-like, regulatory T cell and memory T cell phenotypes were enriched within the antigen-specific IFN-γ-negative CD4 T cell population ^77^. A recent study using collaborative cross mouse strains, a multi-parental genetic reference mouse population derived from eight founder inbred strains that encompasses a high level of genetic diversity, further supports these data by showing that strains presenting T_H_17 transcriptional programs in a low IFN-γ signaling setting were able to control *M. tuberculosis* infection ^78^. Furthermore, a genome wide association study comparing resisters with *M. tuberculosis*-infected individuals revealed a cluster of variants associated with strong protection on chromosome 10q26.2, with predicted functional consequences leading to increased T_H_17 responses in TB resisters ^79^. In another study, TB resistance was associated with MR1-restricted and other donor-unrestricted T cell clonotypes of unknown specificity ^80^, illustrating the complexity and diversity of mechanisms potentially underlying resistance. Recently, single cell RNA-sequencing of bronchoalveolar lavage cells was performed in HIV-infected TB resisters and compared to an LTBI group, revealing extensive changes associated with the resister phenotype ^81^. Specifically, a baseline phenotype of classically activated macrophages and a lymphocyte signature combining cytotoxic genes (granulysin, granzyme B and perforin) and NK receptors were found to be increased in the bronchoalveolar lavage of resisters ^81^. In addition to unique monocytic and T cell responses, a specific antibody profile was reported in TB resisters, with IgM, class-switched IgG antibody responses, enhanced antibody avidity and evidence of T cell help ^82^. Increased CD8 memory T-cell proliferation, higher granzyme B production, and expanded B-cell follicles also correlated with protection from reactivation in the TB/SIV model ^83^. Of note, a contact tracing study in Indonesia reported that a persistently negative IGRA in TB contacts correlated with BCG vaccination, although the protection afforded by BCG decreased with increasing *M. tuberculosis* exposure and age ^84^. Whether this effect of BCG in boosting resistance to *M. tuberculosis* results from trained immunity remains a matter of debate ^85^. Collectively, our current understanding of TB resistance highlights that there are likely multiple immune pathways operating in individuals that do not convert to TST/IGRA positivity, despite high exposure to *M. tuberculosis*.

### The immune response across the TB spectrum: from infection to disease

#### Innate myeloid responses

The primary immune events following *M. tuberculosis* infection, particularly at the macrophage level, are considered to play a determining role in dictating the outcome of infection ^86^ and are informed mainly by experiments performed in mice, since assessing the time of infection in humans is not possible. Sampling infected lung tissue from humans is prohibitive and typically only obtained from postmortems or in cases of therapeutic resection due to advanced or treatment-resistant disease. In mice aerosol-infected with *M. tuberculosis*, the first cells to be infected are alveolar macrophages ^87^, which despite becoming activated and initiating an inflammatory response are permissive to *M. tuberculosis* growth and harbor bacteria displaying a transcriptomic signature of low stress ^88^. Depleting alveolar macrophages or reprogramming them into less permissive cells improved *M. tuberculosis* control in the mouse model of infection ^89, 90^. In addition to being niches for *M. tuberculosis*, alveolar macrophages play a role in the pathogen’s dissemination to the lung interstitium, which was reported to be dependent on IL-1 signaling in the mouse model ^87^. Following the dissemination of *M. tuberculosis* to the lung, several myeloid cells are recruited, including monocyte-derived inflammatory interstitial macrophages. Comparative analyses of these cells with alveolar macrophages showed that interstitial macrophages restrict *M. tuberculosis* growth, display a distinct metabolic profile and impose a stressful environment to the pathogen ^88, 89, 91, 92^.

#### T-cell and NK cell mediated protective responses

The “second” stage of infection, characterized by the establishment of *M. tuberculosis* in the lung and the recruitment of diverse immune cells, likely marks an important bifurcation step leading to control of infection or progression to disease. Control of *M. tuberculosis* and protection from disease development heavily relies on T cell responses (Figure 3b), supported in humans by the observations that immunodeficiency or immunosuppression compromising T cell responses invariably result in TB (discussed above). Despite the absolute requirement of IFN-γ to avoid TB disease, a protective role for T_H_17 cell responses is gaining relevance as these responses were increased in latently infected individuals compared with those progressing to TB ^93^ and in TB resisters ^77^, as detailed above. Conversely, polyfunctional T_H_17-like effector T cells were found to be reduced in individuals who progressed to TB, a reduction that was proposed to precede and persist beyond active disease ^94^ in line with a protective role for T_H_17-cells. *M. tuberculosis*–infected but asymptomatic NHP also displayed increased frequencies of *M. tuberculosis*–specific IL-17+ and IL-17+IFN-γ+ double-positive CXCR3- and CCR6-expressing T cells in the airways ^95^. This is in keeping with a previous study describing the presence of antigen-specific CXCR3+/CCR6+ Th1 cells in latently infected individuals ^96^. Moreover, in infected NHP, granulomas following a trajectory of *M. tuberculosis* growth restriction were enriched in CD4 and CD8 type 1 and 17 cytotoxic T cells, whereas those that failed to control *M. tuberculosis* growth harbored an IFN-activated macrophage population, as well as mast cells, plasma cells and fibroblasts ^97^. However, lung T cells isolated from TB patients displayed residency markers and were enriched for functionally competent IL-17-producing CD4 T cells ^98^. Moreover, IL-17A was enriched in skin recall responses of active TB patients, together with increased neutrophil recruitment and matrix metalloproteinase-1 expression ^99^, suggesting a role in TB pathogenesis.

In other studies, a subset of highly polyfunctional *M. tuberculosis*-specific CD4 T cells defined by CD153 ^100^ was found to be increased in LTBI as compared to active TB cases ^100, 101^, and the frequency of CD153+ *M. tuberculosis*-specific CD4 T cells inversely correlated with bacterial loads in granulomas of infected NHP ^100^ and in TB patients ^101^. Also important, a recent proteome-wide screen of T cell reactivity highlighted the existence of T cell epitopes and antigens specifically enriched in individuals with active versus latent TB ^102^ and lung resident T cells have been shown to highly protective against *M. tuberculosis* in the mouse model ^103^. These data suggest that specific T cell phenotypes promote *M. tuberculosis* control and prevent disease development. T cell epitopes in *M. tuberculosis* are evolutionarily hyperconserved, showing little to no sequence variation, suggesting they are under strong purifying selection rather than immune-driven pressure, presumably to direct T cell responses that favour pathogen survival and dissemination ^104^. While a protective role for T cells during *M. tuberculosis* infections is well established ^26, 33, 105^, their contribution to immunopathology has also been demonstrated in experimental models ^66, 67^. Hence, the antigen type and load, as well as the host microenvironment and the durability of T cell responses may impact whether T cells control infection or cause tissue damage.

Single-cell analyses of *M. tuberculosis*-infected NHP lungs suggested that establishment of latency was accompanied by the accumulation of a CD27+ natural killer (NK) cell subset, which expressed granzyme and perforin and was also detected in the blood of humans with LTBI ^106^. An increase in the abundance of circulating NK cells in LTBI was also found in a study performed across multiple cohorts ^107^.

#### Potential protective effects of B cells and antibodies

The role of B cells in the immune response to *M. tuberculosis* infection is not fully understood and there are various studies in mice which show context dependent results. Heterogeneous, functionally diverse B cell subsets were identified in the lungs of people with TB ^108^, contributing to the mounting evidence in humans indicating that humoral immune responses are protective in TB. Particularly important are the findings that antibody Fc-effector functions diverge across TB disease states ^109, 110^ and the identification of Fc variants with a functional role in restricting *M. tuberculosis* growth ^111, 112^ (Figure 3b). A recent study in mice suggests Fc-independent as well as Fc-dependent mechanisms of antibody-mediated immunity to TB ^113^.

#### Type I IFN and neutrophils dominate detrimental responses

Whole-blood transcriptomics of TB patients revealed a type I IFN-inducible neutrophil driven signature correlating with lung radiographic disease ^114^ which revealed TB heterogeneity ^115^. This signature was diminished upon effective treatment ^13, 116, 117^ and has been validated in many subsequent studies ^93, 116, 118, 119, 120^. Increased blood neutrophil numbers have been independently associated with increased risk of mortality ^121^. Neutrophils are the predominant cell type in the lungs and sputum of TB patients ^122^ and major components of TB granulomas isolated from humans, either deceased, or undergoing lung resections or lymph node biopsies ^123^. Neutrophils, displaying both type I IFN-dependent and independent transcriptional states have very recently been found to be predominant in the bronchoalveolar lavage of a subset of TB patients and in a subset of their household contacts who progressed to TB, closely correlating with *M. tuberculosis* load and extent of lung disease ^7^. Pulmonary neutrophilic inflammation is a common feature in multiple TB-susceptible mouse models, and neutrophil depletion limits disease in these mice ^124, 125, 126, 127, 128, 129^.

Understanding the mechanisms by which type I IFN and neutrophils contribute to TB progression (Figure 3c) remains a topic of intense research. The recapitulation of the human TB immune signatures in certain animal models was suggestive of conserved mechanisms of TB progression and pathogenesis that could be studied in mechanistic experiments in these models further supported by a more recent study whereby immune signatures of pathogenesis in the airways of human TB patients and their contacts who progressed to TB are shown to be recapitulated in published datasets of lung tissue from both NHP and mouse models of TB ^7^. In *M. tuberculosis*–infected TB-susceptible C3HeB/FeJ mice ^131^, blockade of type I IFN receptor (IFNAR1) signaling reduced bacterial growth and disease severity ^128^. Similarly, C57BL/6 mice bearing a susceptibility locus from C3HeB/FeJ mice showed increased TB severity via the action of type I IFN ^132^, as did mice lacking Sp140 which regulates type I IFN ^132, 133^. Mechanistically, type I IFN induced neutrophil extracellular trap (NET) formation in *M. tuberculosis*– infected C3HeB/FeJ mice ^128^ and drove neutrophil swarming in TB lesions, impeding T cell-macrophage interactions ^124^. High susceptibility to *M. tuberculosis* infection in mice lacking the *Atg5* gene specifically in macrophages, monocytes, neutrophils and some DCs, was also mechanistically related to type I IFN-induced neutrophil swarming and NET formation^134^. These findings in experimental mouse models support data where neutrophils were shown to promote *M. tuberculosis* growth and to contribute to tissue damage in human TB ^128, 135^ and in NHP ^136^. During established disease in highly susceptible Sp140-deficient mice, plasmacytoid dendritic cells (pDCs) and interstitial macrophages have been suggested as the main sources of type I IFN in mice, and an accumulation of pDCs was reported in proximity to TB granulomas in humans and NHP ^133^. Furthermore, in NHP the influx of pDCs and of a macrophage subset responsive to IFN and activated T cell responses correlated with pulmonary TB disease ^106^. In support of such a mechanism, it has been reported that neutrophil-pDC interactions lead to production of type I IFN in response to *M. tuberculosis* ^137^.

More recently, it has become evident that the spatial location of neutrophils within TB lesions hinders the interactions of protective CD4 T cells with infected macrophages, thus impairing control of *M. tuberculosis* ^124, 125^. This is relevant as previous studies showed that the correct positioning of CD4 T cells within the granuloma and their interaction with infected macrophages is critical for protection ^138, 139^, as adaptive immunity fails to effectively restrict *M. tuberculosis* growth within granulocytes ^140^. The intricate relationship between neutrophils and T cells is further illustrated by the fact that T cell responses, in particular IFN-γ-producing T_H_1 cells, are necessary for controlling neutrophils and preventing pathology ^129^. Moreover, TB-resistant C57BL/6 mice or mice with prior T cell immunity, exhibited limited neutrophil driven-pathology in TB lesions ^124, 125^, suggesting that timely and appropriately located T cell-macrophage interactions may overcome the detrimental effects of activated neutrophils in the lungs. However, the role of neutrophils in TB is possibly context-dependent, since a TB household contacts study showed an inverse correlation between neutrophil counts in the blood and the risk of infection possibly reflecting a role in innate immunity ^141^. Also, in mice, neutrophils were proposed to impact the efficacy of DCs in initiating T cell activation, and consequently to promote adaptive immune responses to *M. tuberculosis* ^142^.

### The genetic diversity of *M. tuberculosis* impacts host-pathogen interactions and TB outcomes

**Figure 4:**
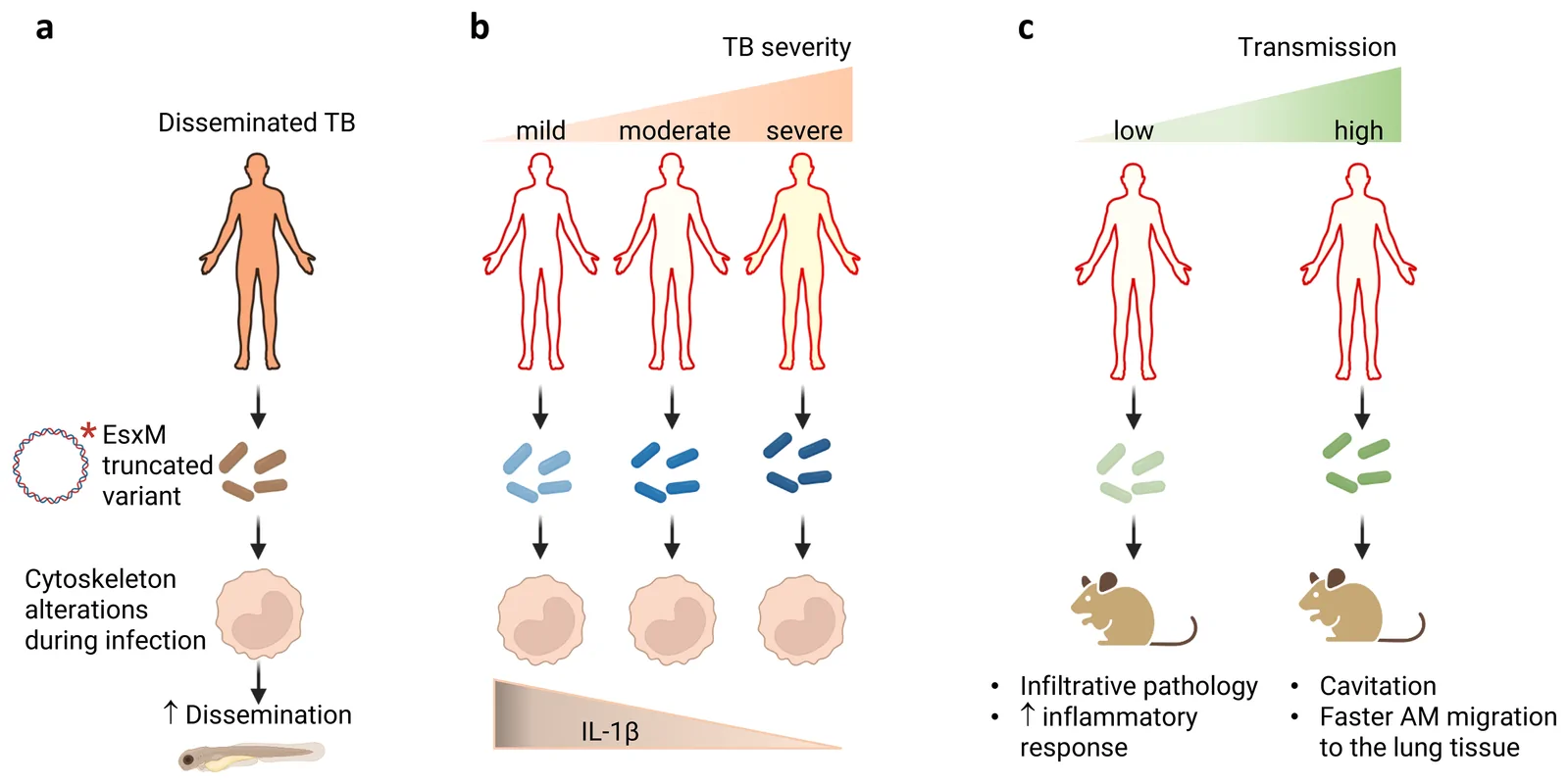
Contribution of *M. tuberculosis* heterogeneity and differential host interactions to distinct TB clinical manifestations. The study of genetically diverse *M. tuberculosis* isolated from patients with different TB manifestations is revealing bacterial modifiers of TB disease and novel mechanisms of pathogenesis. **a**. *M. tuberculosis* causing an outbreak of disseminated TB was found to carry an ancestral full-length version of the type VII-secreted effector EsxM rather than the truncated version present in other modern *M. tuberculosis* lineages. The ancestral EsxM variant altered the macrophage actin dynamics and associated with increased dissemination and bone disease in zebrafish models ^150^. **b**. In a cohort of pulmonary TB patients stratified for disease severity, *M. tuberculosis* strains isolated from mild TB cases led to increased inflammasome activation and IL-1β secretion by human and mouse macrophages, a mechanism that was evaded by severe-TB causing strains ^151^. **c**. *M. tuberculosis* strains isolated from TB index cases classified as high or low transmitters in a household contact study led differential immune responses and pathology in infected C3HeB/FeJ mice, with strains associated with high transmission promoting a faster, IL-1R-dependent migration of alveolar macrophages from the airway to the tissue, with formation of cavitated lesions at later time-points post-infection ^153, 154^. *AM, alveolar macrophage; TB, tuberculosis*.

As discussed earlier, the genetic diversity of *M. tuberculosis*, as compared to other pathogens, is very low ^6^ and a whole-genome sequencing analysis study suggested that successful *M. tuberculosis* clones need not be intrinsically exceptional and that host, environmental, and social determinants likely play major roles in sustaining transmission in high-incidence settings ^143^. Despite these findings, the lengthy co-existence of *M. tuberculosis* with its human host has shaped the pathogenesis of TB, with a growing body of evidence demonstrating that *M. tuberculosis* lineage-specific variants impact transmissibility, immune responses and disease outcomes ^144, 145^. The biology underlying how genetically related *M. tuberculosis* clinical isolates modulate host immune responses to drive disease of varying severity remains poorly understood. However, it is evident from existing studies that several strategies have been developed by *M. tuberculosis* to this end. One such strategy is the enhanced induction of type I IFN by certain *M. tuberculosis* isolates, which consequently worsened outcomes of infection in the mouse model ^128, 131, 146, 147^. Another strategy relies on the production of a polyketide synthase-derived phenolic glycolipid by a group of *M. tuberculosis* isolates of Lineage 2 (W-Beijing) which inhibited the inflammatory responses of monocyte-derived macrophages and was associated with hypervirulence in mice ^148^ and rabbits ^149^. More recently, significant advances have been made by linking clinical manifestations of TB with functional analyses of *M. tuberculosis*-host interactions. Isolating the strain of *M. tuberculosis* causing a TB outbreak characterized by high rates of disseminated TB and skeletal disease and sequencing its genome led to the identification of an EsxM variant which altered the macrophage cytoskeleton, thus favouring dissemination ^150^ (Figure 4a). This mode of action was further validated using *Mycobacterium marinum* in the zebrafish model of infection ^150^. Furthermore, the study of *M. tuberculosis* strains isolated from a cohort of TB patients stratified for disease severity revealed that isolates recovered from people with severe TB evaded macrophage cytosolic surveillance systems, including the inflammasome, to reduce the secretion of IL-1β ^151^ (Figure 4b). Such *M. tuberculosis* isolates have also been reported to accumulate mutations in the genes encoding components or regulators of the Esx secretion system, although the genetic basis underlying this phenotype remains under investigation ^151^. On the other hand, a lineage 3 *M. tuberculosis* isolate from an outbreak in the UK revealed absence of FadB4, a protein with a putative role in cell envelope biosynthesis, as a mechanism possibly associated with increased IL-1 responses in macrophages and cavitary disease and transmission in humans ^152^. Also linked to the modulation of IL-1 responses, *M. tuberculosis* isolates associated with high transmission to household contacts, based on high proportion of detected TST positivity, induced faster IL-1R-dependent alveolar macrophage migration from the alveolus into the lung interstitium in the mouse model of infection ^153^. This subsequently promoted early dissemination of bacteria to the lymph nodes, T_H_1 priming, bacterial control and granulomatous responses ^153^, which were previously shown in the mouse model to have a higher potential to cavitate than those caused by *M. tuberculosis* isolates selected from low transmission settings ^154^ (Figure 4c). Interestingly, transmissibility was further associated with macrophage responses in a study showing that geographically restricted lineages of the *Mycobacterium tuberculosis* complex (MTBC) display lower infectivity in macrophages from unrelated (allopatric) populations, which may explain their lower transmissibility in these hosts ^155^. A recent high throughput phenogenomics analysis further supports the idea that *M. tuberculosis* genotypes dictate clinically relevant TB outcomes, including cavitary disease ^156^. Thus, while there is overall low genetic variation in *M. tuberculosis*, this finding is functionally significant and currently considered an important variable in TB research ^157^. A growing body of evidence shows that in addition to genetic variations in the host or *M. tuberculosis*, human genome-*M. tuberculosis* genome interactions also determine TB manifestations and immune responses as shown in a number of studies and in human populations ^158, 159, 160, 161^. The host gene variants found in the referenced studies encode proteins with a role in the immune response, demonstrating the strong interaction of *M. tuberculosis* variants with host immunity. Of note, the outcome of infection in the mouse model is also shaped by host and pathogen genomes ^162, 163, 164^, with the combination of a specific mouse genetic background (C3HeB/FeJ) and a clinical *M. tuberculosis* isolate (HN878) shown to better recapitulate the whole-blood transcriptional signature widely detected in human TB patients ^131^.

### TB disease outcomes depend on the dose and frequency of infection

In high endemic settings it has been suggested that cough may not always be required for *M. tuberculosis* transmission, with release and exposure to the pathogen likely occurring in a persistent form ^165^. Therefore, it is conceivable that the dose and frequency of exposure are not similar in all individuals. Addressing the effect of the dose of infection in humans is very difficult, but some studies provide evidence that it plays a role in the risk of infection and disease ^166, 167^. Experimental TB models support the relevance of the infection dose to disease outcome. In rabbits the dose of infection was shown to impact bacillary loads at different time-points post-infection, with higher lung bacillary loads observed with increasing doses of inoculum ^168^. In mice, an effect of the infection dose was seen in the whole-blood transcriptional immune signatures of TB disease, with over-abundant type I IFN modules showing a graded increase from low to high doses of infection ^131^. Finally, ultra-low dose infections in mice have been reported to result in tissue lesions that shared features with human granulomas ^169^.

Reinfection with *M. tuberculosis* occurs in high burden settings ^14, 170, 171, 172^, and advances in *M. tuberculosis* whole genome sequencing applied to human samples unequivocally show that multiple infections are frequent, and currently underestimated, in these settings ^173^. From a clinical perspective, reinfection with *M. tuberculosis* elevates the risk of progression to TB disease ^174, 175^ and in some settings has been associated with poorer outcomes of disease ^176, 177^. The increased risk of TB and of poorer TB outcomes in individuals who have previously suffered from TB is also probably related to persistent lung damage despite microbiological cure, a condition generally termed post-TB lung disease ^178^. Reinfection also bears implications for post-primary TB, as in this case *M. tuberculosis* encounters a host that is no longer immunologically naïve to it. From an immune perspective, reinfections, as well as infections with different *M. tuberculosis* strains (i.e. mixed infections), raise the question of whether and how an initial *M. tuberculosis* infection may protect from a subsequent one. Studies in NHP show that ongoing *M. tuberculosis* infection provides robust protection against secondary infections ^179^. Although this is much more difficult to assess in humans, a previous study showed that individuals with LTBI had up to 80% lower risk of developing TB after reinfection than uninfected individuals ^180^. Mathematical modelling data from NHP and the pre-antibiotic era in humans led to the hypothesis that exposure to *M. tuberculosis* infection promotes concomitant immunity via the accumulation of antigen-specific tissue resident memory T cells ^181^. These predictions align to some extent with experimental data in the NHP model of re-infection, which revealed that the presence of CD4 T cells during reinfection following antibiotic treatment of a prior infection was essential for protection ^182^. Protection was accompanied by changes in CD8 T cell phenotype, reduced neutrophilia, and regulated myeloid cell responses ^182^. Although prior infection confers some level of protection as shown in these NHP studies, this immune protection is not sterilizing ^179, 182^. Despite these advances, it remains unclear whether pre-existing immunity is long-lasting, equally efficient towards infections across all *M. tuberculosis* strains and how it may change the structure of granulomas. In this context, a recent study based on the mouse model of concomitant immunity showed that the early recruitment of T cells upon re-infection both activates infected macrophages to promote *M. tuberculosis* control and counter-regulates detrimental neutrophil responses to avoid tissue damage, thus profoundly changing the architecture of lesions ^125^.

### Challenges for TB immunology research

Despite the advances in our understanding of the mechanisms underlying the immune response in TB there are multiple questions remaining. A substantial challenge to the field is to translate those advances into the development of an effective TB vaccine. The incomplete protection observed following natural *M. tuberculosis* infection of NHP ^179, 182^ suggests that efficacious vaccines may require appropriate combinations of *M. tuberculosis* antigens, adjuvant and routes of immunization to drive protective responses superior to natural infection. Although IFN-γ is undoubtedly required for protection against TB, this cytokine has not proved to be a good correlate of protection in TB ^183^. Moreover, as discussed above, it is increasingly appreciated that excessive IFN-γ is detrimental for TB outcomes ^65, 66, 67^ and that IFN-γ-independent mechanisms contribute to protection against TB ^26, 33, 105, 184^. Furthermore, it is possible that not all *M. tuberculosis* antigens induce IFN-γ responses, but instead for example T_H_17 responses in humans, as recently described (pre-print ^185^). Advances in immunopeptidomics combined with high-throughput functional technologies applied to human *M. tuberculosis-*infected cells are already contributing to unveiling novel candidate antigens and strategies for TB vaccine design ^186, 187^. Potentiating humoral responses also deserves attention, as they have been linked to vaccine efficiency in humans ^176^ and NHP ^188, 189^. The development of new TB vaccines will require careful consideration of the complexity of host-*M. tuberculosis* interactions that shape the immune response. This is readily seen in the mouse model, where vaccination outcomes depended on the mouse genetic background and also on the infecting *M. tuberculosis* strain ^162, 190^. That the choice of vaccine and route of administration may also be critical to induce the appropriate protective immune response is supported by the findings in NHP, showing that intra-venous BCG vaccination as opposed to classical intra-dermal route can provide enhanced protective immunity. Intravenous BCG immunization was associated with greater antigen-responsive CD4 and CD8 T cell systemic and local pulmonary responses ^191^, and required both CD4 and CD8 T cell subsets ^192^. Subsequent studies identified airway antigen-specific CD4 T cells and NK cells ^193^, polyfunctional T cells with strong T_H_1 and T_H_17 responses following purified protein derivative (PPD) stimulation ^194^ and NK cells expressing granzyme B but not IFN-γ ^195^, as strong correlates of protection. Finally, given that innate immune cell populations also undergo functional adaptation after infection or vaccination and develop an innate memory program, coined trained immunity, exploring this mechanism for vaccine development is an exciting area ^196^.

The importance of understanding the immune response in the lung and draining lymph nodes in situ and how this relates to infection outcome is also a challenge for future TB immunology research. It is likely that T cells need to interact directly with infected macrophages to mediate protective functions, which remain incompletely defined. Immune cells with similar apparent phenotypes could be protective or detrimental depending on their location and timing of priming and action. Spatial analyses, including spatial transcriptomics and high-dimensional tissue immunofluorescence techniques, at different stages after infection will help reveal how initial lesions progress to form the granuloma and how perturbations that affect infection outcome impact the spatial interactions of immune cells in the lung. A growing number of studies using different techniques to dissect the granuloma structure in human active TB biopsies, have revealed distinct regional immune microenvironments ^123, 197, 198, 199, 200, 201^. Similarly, studies performed in NHP ^202^ and mice ^203^ highlight the heterogeneity of the immune microenvironments in lung lesions from *M.tuberculosis* infected animals ^125, 138, 201^. Elucidating this spatial organization of immune cells and effector molecules in the developing granuloma and linking these events to infection outcome will require in depth perturbation studies in experimental systems. Studies in TB-susceptible mice have recently shown that swarming neutrophils in TB lesions functionally oppose T cell-macrophage interactions and contribute to failed early control of *M. tuberculosis* infection ^124, 125^. Prevention of aberrant neutrophil responses by blockade of their migration into lesions and/or their activation by type I IFN or other type I IFN-independent pathways may have promise as host-directed therapies. Further spatial interrogation of cell-cell and ligand-receptor interactions within lung TB lesions over the course of infection may reveal additional immune molecules and pathways which correlate with protection or disease progression, to test as potential targets for host-directed therapies aimed at unleashing the full potential of protective T cells.

## Conclusions

TB remains a major public health concern and its control heavily relies on the development of more effective vaccines, better diagnosis and host-directed therapies that can overcome antimicrobial resistance. While a deeper understanding of the immune response to infection with *M. tuberculosis* that results in protection or progression to TB disease will help to fill these gaps, it is critical to consider the complexity of the events determining infection outcome. Firstly, the study of how the immune response to *M. tuberculosis* unfolds across the complex spectrum of TB outcomes. Secondly, the integration of the pathogen’s diversity, how it evades immune responses and its contribution to TB manifestation. Finally, the use of systems immunology approaches allowing a full vision of the immune response across the spectrum of TB outcomes will undoubtedly contribute to TB control.

## Figures and Tables

**Table 1:** Host conditions that increase the risk of TB disease.

Condition	Impact	Reference
Age	Infants: higher risk of infection; typically presenting with extracellular forms of TB, most notably milliary TB and tuberculous meningitis. Adults: pulmonary TB becomes the most prevalent form of disease. Elderly: TB presentation resembling that of HIV-TB, with more disseminated forms and less cavitation, likely due to immunosenescence particularly of the CD4 T cell compartment.	^1, 59, 60^
Sex	A larger proportion of TB cases is reported in men. An effect of different sex hormones on the immune system is possible. For example, alterations in the levels of cortisol may impact the production of IFN-γ in response to Mycobacteria, although differences in lifestyle cannot be discounted.	^1, 61^
Diabetes mellitus (DM)	Increased cavitary lung lesions, higher lung mycobacterial burdens, longer periods of infectiousness and worse outcomes. Broad immune dysfunction with impaired innate and adaptive immune responses described in TB-DM patients.	^62^
Silicosis	Increased risk of TB in individuals exposed to silica. Mechanisms not fully understood. Aberrant production of type I IFN with predominant Th2 responses were described in the mouse model, in the lung upon silica exposure, which compromised the host ability to limit *M. tuberculosis.*	^63, 64^
Anti-TNF therapies	TB reactivation/infection in patients receiving TNF blockers. TNF is required for macrophage respiratory burst in response to *M. tuberculosis* infection.	^36, 50^
Anti-PD1 therapies	TB episodes associated with high mortality in cancer patients receiving PD1 blockade immune therapy. Validated in mouse and NHP models. PD-1 blockade boosts T_H_1 cell function, resulting in in increased inflammatory responses and TB exacerbation in mice and NHP.	^65, 66, 67, 68^
